# Pathogenomics for accurate diagnosis, treatment, prognosis of oncology: a cutting edge overview

**DOI:** 10.1186/s12967-024-04915-3

**Published:** 2024-02-03

**Authors:** Xiaobing Feng, Wen Shu, Mingya Li, Junyu Li, Junyao Xu, Min He

**Affiliations:** 1 College of Electrical and Information Engineering, Hunan University, Changsha, China; 2 Present Address: Zhejiang Cancer Hospital, Hangzhou Institute of Medicine (HIM), Chinese Academy of Sciences, Hangzhou, 310022 Zhejiang China

**Keywords:** Pathogenomics, Pathomics, Genomics, Computational pathology, Precision oncology

## Abstract

The capability to gather heterogeneous data, alongside the increasing power of artificial intelligence to examine it, leading a revolution in harnessing multimodal data in the life sciences. However, most approaches are limited to unimodal data, leaving integrated approaches across modalities relatively underdeveloped in computational pathology. Pathogenomics, as an invasive method to integrate advanced molecular diagnostics from genomic data, morphological information from histopathological imaging, and codified clinical data enable the discovery of new multimodal cancer biomarkers to propel the field of precision oncology in the coming decade. In this perspective, we offer our opinions on synthesizing complementary modalities of data with emerging multimodal artificial intelligence methods in pathogenomics. It includes correlation between the pathological and genomic profile of cancer, fusion of histology, and genomics profile of cancer. We also present challenges, opportunities, and avenues for future work.

## Background

Pathogenomics is an innovative imaging analysis method that leverages invasive techniques to draw correlations between genomics with pathological image features. This approach provides a more profound comprehension of tumor biology and allows for the capture of the inherent heterogeneity of tumors. The ultimate objective of pathogenomics is to develop specific imaging biomarkers that combine genotypic and phenotypic metrics.

With an estimated 19.3 million new cancer cases and nearly 10 million cancer deaths occurred worldwide in 2020 [1], innovation in cancer diagnosis and treatment is desperately needed. Cancer diagnosis and prediction of treatment and prognosis often harness heterogeneous data resources, including whole slide images (WSI), molecular profiles, and clinical data such as patient age and comorbidities [2, 3]. Several recent studies have illustrated that patterns found in high-dimensional, multimodal data can improve prognostication of disease invasiveness, stratification, and patient outcomes compared to unimodal data [2, 4–7].

As the field of anatomical pathology moves from slides to digitized whole slide images, and the breakthrough of next-generation sequencing (NGS), alongside rapid progress via deep learning and other advanced machine learning methods has been made in each of individual modalities, major unsolved questions about how to take advantage of multimodal data for integration and mine useful information remain. Thereby, this is a critical opportunity to develop joint histopathology-genomics analysis based on artificial intelligence (AI) approaches that leverage phenotypic and genotypic information in an integrated manner, that is, pathogenomics.

At present, the analysis of histopathological imaging is still mainly in the stage of manual way [2, 8–10], supplemented by computer quantitative analysis. Through great progress made by quantitative analysis at this stage, the analysis mainly studies a few common features, such as morphological features, color and texture features, shape features, etc., while use single feature cannot cover the complexity and variability of tumors, leaving an urgent problem to be solved in quantitative analysis of computational pathology [11]. Subjective and qualitative histopathology-based image analysis of the tumor microenvironment (TME), alongside with quantitative examination of omic analysis, especially genomics, is the standard-of-care for most cancers in modern clinical practice [3, 5, 6, 12–15]. Tumor microenvironment is the complex cellular environment that mainly composed of blood vessels, extracellular matrix (ECM), immune cells, fibroblasts, inflammatory cells, and various signaling molecules around the tumor [16]. Although histological tissue analysis provides significant morphological and spatial information on the TMI, with different inter- and intra-observer variability qualitatively examined by experienced pathologists, interpretation at the histological level alone can hardly take advantage of abundant phenotypic information that has been shown to correlate with prognosis.

Genomic analysis focuses on monitoring cellular activity at the molecular level, compared to pathology quantifies disease phenotypes. Current modern sequencing technologies, such as single-cell sequencing, can parse the genomic information of a single cell in tumor specimens, while spatially resolved transcriptomics and multipath immunofluorescence technologies can simultaneously parse histopathological morphology and gene expression in space [17–21]. Bulk sequencing can reveal the presence and quantity of all genes and TME in tumors within a given time, to help us understand the molecular discrepancy between disease phenotypes and responses to treatment. Genomic analysis of tissue biopsies can provide quantitative information on genome expression and alterations, including gene mutations, copy number changes (CNV), and DNA methylation, but it is challenging to recognize tumor-induced genotype measurements and alterations via no-tumor entities such as normal cells.

**Fig. 1:**
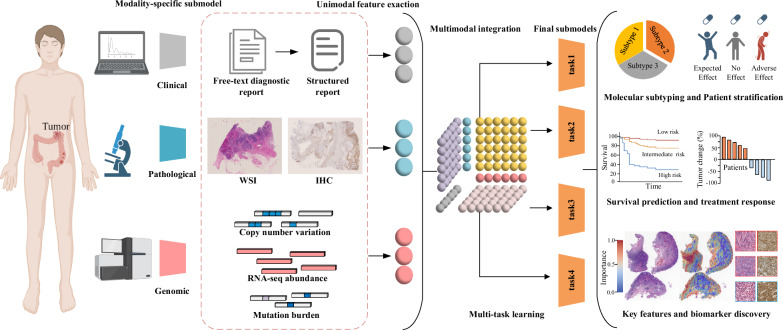
Example data modalities for multimodal integration include clinical, pathological, and genomic profiles. Submodels extract unimodal features across each data modality. Then, a multimodal integration step generates intermodal features—a tensor modal fusion network. And final sub-models infer multi-task learning in patient outcomes and clinical prognostication, including patient stratification and molecular subtyping, survival prediction and treatment response, discovery of key features and novel biomarkers. (Created with BioRender.com.) *WSI* whole slide image, *IHC* immunohistochemistry

Therefore, the emergence of pathogenomics provides an exciting opportunity to combine morphological information from histopathology and molecular information from genomic profiles to better quantify the tumor microenvironment and harness advanced machine learning algorithms for the discovery of potential histopathology-genomics based biomarkers and precision oncology in accurate diagnosis, treatment, and prognosis prediction. To exemplify this premise of pathogenomics, we will focus on three major modalities in cancer data: histopathology, genomics profile, and clinical information (Fig. 1).

The integration of histopathological phenotypes and genotypes could help us:

1. Understand context-aware linkage between tissue architectures and molecular properties;
2. Capture more “understandable” spatial features through systematic and quantitative analysis;
3. Discover novel diagnostic and prognostic image-omics-based biomarkers;
4. Gain complimentary information for visualizing pathological and molecular context of cancer;
5. Develop multimodal fusion models for prognostication of survival situation, gene mutation signatures, patient stratification and treatment response.

In this work, we provide a brief review of representative works that focus on integrating pathomics and genomics for cancer diagnosis, treatment, and prognosis, including the correlation of pathomics and genomics, fusion of pathomics and genomics. We also present challenges, potential opportunities, and perspectives for future work. An overview of the fusion of pathomics and genomics analysis is shown in Fig. 1.

## Correlation between pathological and genomic profile of cancer

Correlating pathological morphology with large-scale genomic analysis has become a burgeoning field of research in recent years [8, 22]. Given that most WSIs currently lack pixel-level annotations, it can be verified to a certain extent whether it is consistent with known biological mechanisms by correlating image features with molecular data. For instance, to explore whether image immune features are related to immune regulatory genes, to further validate whether the image features obtained by machine learning algorithms are reliable to replace the doctor’s manual estimation in the future. Moreover, the association analysis of image features and molecular expression patterns can bring new inspiration to cancer biology research and help to find potential new biomarkers [23].

### Whole slide images and computational pathology

Whole slide images offer a wealth of pathological information, including details about nucleus shape, texture, global structure, local structure, collagen pattern, and tumor-infiltrating lymphocytes (TILs) pattern. However, the high complexity of WSIs, due to their large size (a resolution of 100k × 100k is common), presence of color information (hematoxylin and eosin and immunohistochemistry), no apparent anatomical orientation as in radiology, availability of information at multiple scales (e.g., 4×, 20×), and multiple z-stack levels [11], make it challenging for human readers to precisely extract such visual information. Fortunately, the advent of artificial intelligence and machine learning tools in digital pathology enable mining histopathological morphometric phenotypes and might, ultimately, improve precision oncology.

Stained tissue samples, observed under an optical microscope, provide detailed insights into the morphological distribution of cells in different biological states, as well as the composition of the tumor microenvironment (TME). Whole slide image allows clinicians to analyze and evaluate these varied aspects of the TME, such as tissues and cells of cancer patients. This analysis can help identify the benign or malignant nature of cancer, classify the tumor grade, extent of invasion, and prognosis, leading to a qualitative or semi-quantitative diagnosis. As a result, WSI is currently considered the “gold standard” for clinical diagnosis of cancer. However, given the high self-heterogeneity of tumors, manual estimation on images can be subjective and its accuracy can be affected by the pathologist’s own clinical experience and working conditions. This can lead to inevitable human bias, resulting in disagreement in diagnosis or even misdiagnosis. In recent years, with the rapid development of machine learning, more and more studies have started applying advanced algorithms to WSIs to automatically identify and quantitatively analyze important tissues and cells in images, thereby assisting clinical evaluation and related computational pathology studies. Colorectal [24–26], breast [27, 28], gastrointestinal [29, 30], prostate [31, 32], and lung cancers [33–35] can be retrieved by automatic classification or quantitative analysis in advanced machine learning algorithms using multicenter, large cohort WSI data.

### Computational pathology reveals prognostication of gene mutations, cancer subtypes, stratification, and prognosis

The causal and inferential relationships between gene expression and pathology are indeed crucial in the discovery of biomarkers. Hematoxylin–eosin (H&E)-stained WSIs and immunohistochemistry (IHC) data have been leveraged to predict molecular features of tumors and to discover new prognostic associations with clinical outcomes. We refer readers to several well-chosen extraordinary reviews in these areas [2, 8, 11, 36].

One notable multicenter example in pan-cancer showed that deep residual learning (Resnet 18) can predict microsatellite instability (MSI) status directly from H&E histology [37, 38], suggested a convenient and effective way to identify biomarker for response to immune checkpoint blockade (ICB). Similarly, deep-learning models can assist pathologists in the detection of cancer subtype or gene mutations [24, 33]. However, these deep learning methods rely heavily on large training cohorts and suffer from poor interpretability analysis, thousands of hand-crafted features or manual pixel-level annotations are often needed to achieve excellent, generalizable performance depending on task and data complexity. Harnessing different data modalities at a large clinical-grade scale often requires reducing this burden of time-consuming annotation, especially in multimodal tasks.

Interpretable quantitative analysis of histological images can also be performed using weakly supervised learning without tissue annotation, identifying biological features such as TILs and other properties of the TME and their correlation with molecular features. A recent study found that HE2RNA [39], a model based on the integration of multiple data modes, can be trained to systematically predict RNA-Seq profiles from whole-slide images alone, without expert annotation. The proposed model HE2RNA can be applied for clinical diagnostic purposes such as the identification of tumors with MSI. And other studies [33, 40–42] have linked biologically interpretable features with clinical outcomes, which have revealed gene mutations, tumor composition, and prognosis. Table 1 summarizes the representative research works that relate to pathomics and genomics correlation.

**Table 1:** Overview of research works on correlating pathomics with genomics

Tumor type	Models	References	Data modalities	Performance
HGSOC	ResNet-18, Cox	Kevin et al. [41]	WSI, CT, CE-CT, and NGS	Survival prediction, C-index: 0.61
BRCA and LUAD	Inception v3	Alona et al. [42]	WSI, RNA, and miRNA,	miR-17 status prediction, AUC: 0.95
Pan-cancer	Shufflenet, densenet, inception, and resnet	Jakob et al. [14]	WSI and CNV	MSI status prediction, AUC: 0.89
LUAD	ResNet18, GO, and KEGG	Yi et al. [53]	WSI, mRNA, and clinical characteristics	TMB status prediction, AUC: 0.64
Pan-cancer	HE2RNA	Benoît et al. [39]	WSI and RNA-Seq	The model could predict RNA-Seq profiles from WSIs
STAD	ResNet18, AlexNet, vgg19, squeezenet	Jakob et al. [38]	WSI, MSI and clinical information	Shown that deep residual learning can predict MSI directly from H&E histology
LUAD and LUSC	Inception v3	Nicolas et al. [33]	WSI and gene mutation data	3-Class classification performance reached AUC: 0.97; gene mutations AUCs from 0.733 to 0.856
Pan-cancer	Deep transfer learning, inception V4, COX	Yu et al. [13]	WSI, genomics, transcriptomics and survival data	Shown that deep learning could characterize the molecular bias of tumor pathology
COAD and READ	GCN	Ding et al. [54]	WSI and gene mutation data	Shown that GCN models could predict molecular profile from WSIs
KIRC	LR, RF, SVM, Adaboost	Zheng et al. [55]	WSI and DNA methylation	Shown that ML algorithms can associate the DNA methylation with histological features

*DL* deep learning, *HGSOC* high-grade serous ovarian cancer, *CT* computed tomography, *CE-CT* contrast-enhanced computed tomography, *BRCA* breast cancer, *LUAD* lung adenocarcinoma, *STAD* stomach cancer, *LUSC* lung squamous cell carcinoma, *CRC* colorectal cancer, *KIRC* kidney clear cell carcinoma, *TNBC* triple-negative breast cancer, *CNN* convolutional neural network, *WSI* whole slide image, *KEGG* Kyoto Encyclopedia of Genes and Genomes, *GO* Gene Ontology, *C-index* concordance index, *NGS* next generation sequencing, *RF* random forest, *MSI* microsatellite, *AUC* area under curves, *mRNA* messenger-RNA, *miRNA* micro-RNA, *CAMS* the Chinese Academy of Medical Sciences, China, *NLST* the National Lung Screening Trial, *SPORE* the University of Texas Special Program of Research Excellence, *LR* logistic regression, *SVM* support vector machines, *RF* ransom forest, *TCGA* The Cancer Genome Atlas, *COAD* colon adenocarcinoma, *READ* rectum adenocarcinoma, *GCN* graph convolutional network, *KIRC* kidney renal clear cell carcinoma, *ML* machine learning

Molecular signatures are the most intuitive measurements of response to therapeutic interventions, while survival analysis for cancer prognosis prediction is a standard approach for biomarker discovery, stratification of patients into different treatment groups, and therapeutic response prediction [43]. Many studies have explored the relationship between pathological phenotypic characteristics and cancer prognosis prediction. The histopathological boundary morphology and spatial distribution morphological characteristics extracted based on different machine learning models have predictive effects on cancer grading and prognosis. Recent work has incorporated deep learning into survival analysis, with common covariates including CNV, mutation status, and RNA sequencing (RNA-Seq) expression [44, 45], to examine the relationship between gene signatures and survival outcomes. Nevertheless, these survival analysis for cancer outcome prediction is mainly based on genomic profiles, lack of leveraging heterogeneous information from the inherent phenotypic data sources, including diagnostic slides, IHC slides, which has known significant prognostic value.

Therefore, some studies further combine gene expression data at the molecular level with histopathological features to improve the accuracy of prognosis prediction. For instance, Savage et al. [46] found that combining gene expression, copy number variation, and pathological image features could improve chemotherapy efficacy and prognosis prediction in breast cancer. Cheng et al. [47] found that combining morphological features of cells in pathological images with feature genes of functional genetic data can lead to better prognosis prediction of renal cancer than using image data or genetic data alone. Mobadersany et al. [48] first proposed a deep learning-based framework to predict the prognostic ability of pathological image features, and then extended this model to unify image features with genomic biomarker data to predict glioma prognosis.

Correlation between histopathological and genomic profiles found that patients have a better prediction of prognosis [30, 49–51], but further refinement is needed to better address clinically meaningful subgroups. Emerging spatial genomics techniques [7, 15, 18, 52] and complementary clinical and imaging modalities are opportunities to enrich these data and refine prognostication.

## Fusion of histology and genomic profile of cancer

**Fig. 2:**
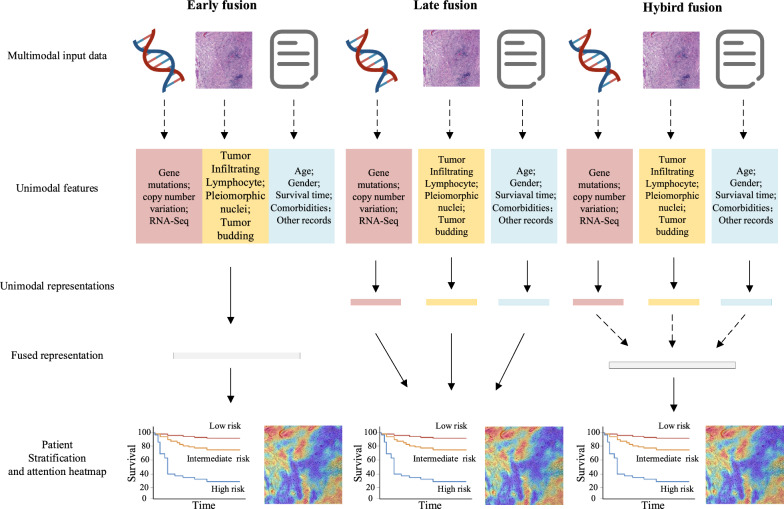
Fusion strategies for multimodal models with genomic profiles, histopathological images, and clinical information. Solid arrows denote stages with learnable parameters (linear or otherwise), dashed arrows denote stages without learnable parameters, and dashed and dotted arrows denote the options for learnable parameters, depending on the model architecture

Complementary information from combining these various modalities, including the morphological and spatial information from digital pathology, and the molecular changes underpinning them, and the corresponding structured pathology reports, is already accelerating biological discovery and applied multimodal tools research in cancer. We suggest that such unimodal models across histopathology, molecular, and clinical domains discussed above will become the building blocks of integrated multimodal models for pathogenomics. The design choices for multimodal models in pathogenomics are shown in Fig. 2.

Understanding the histological context of genomic data is indeed essential for a comprehensive understanding of a tumor’s clinical behavior. Given the intra-tumor heterogeneity, the expression level of certain genes may vary significantly across different regions within the same tumor. Besides, the diagnostic slide of tissue samples provides a global view of tumor morphology, and thus pathomic analysis could alleviate the sampling issues raised in genomic analysis. However, relying solely on pathological features or genomic expressions may not be able to provide biological explanations for some clinical behaviors. Therefore, many researchers have attempted to combine these two data modalities to create more accurate and reasonable diagnostic companion tools.

### Multimodal fusion approaches

The increasing availability of biomedical heterogeneous data modalities, such as electrical health records, medical images, and multi-omics sequences, has paved the way for the development of multimodal intelligence solutions that capture the complexity of human health and disease [56, 57].

These solutions utilize model-agnostic fusion methods, which means that multimodal fusion does not directly rely on specific machine learning methods [27]. Model-agnostic approaches can be divided into early fusion, late fusion, and hybrid fusion [58].

#### Early fusion

Early Fusion refers to the process of combining the original data of different modes or the learned private features of each mode representation before they are fed into a machine learning model. Since early fusion is feature-based, it can ensure that low-dimensional information, such as spatial features of each modality, is preserved during fusion. This method is suitable for each modality that does not have complete information to complete the target task independently. In other words, the fusion decision in early fusion is directly made based on the original data or the learned private features of each mode representation. This fusion method can be expressed as Eq. (1):

1 $${y = D\left( {F\left( {x_{1} ,x_{2} , \ldots ,x_{n} } \right)} \right)},$$

where $x_{i}$ denotes *i*-th data source, $f\left( {x_{i} } \right)$ represents the feature extracted by the model, $F$ represents the feature fusion method, and $D$ represents the decision method made by the model based on the fusion feature.

#### Late fusion

In late fusion, each modality’s data is processed separately and the results are then combined to make a final decision. This approach allows each modality to focus on the features that are most relevant to it, which can improve the accuracy of the final decision. However, it requires that each individual model be highly accurate, as the final prediction is dependent on the accuracy of all individual models. This fusion method can be expressed as Eqs. (2) and (3):

2 $${y = D\left( {d\left( {x_{1} } \right),d(x_{2} ), \ldots ,d(x_{n} )} \right)},$$

3 $${d\left( {x_{i} } \right) = M_{i} \left( {f\left( {x_{i} } \right)} \right)},$$

where $x_{i}$ denotes *i*-th data source, $f\left( {x_{i} } \right)$ represents the feature extracted by the model, $d(x_{i} )$ represents the decision judgment made by the model $M_{i}$, and $D$ represents the comprehensive decision method.

The commonly used comprehensive decision-making methods in late fusion include: (1) average fusion: the final prediction is made by averaging the confidence score of the output of the multimodal model; (2) weighted fusion: the final decision is made by calculating the comprehensive confidence score by weighting the output confidence of the multimodal model [59].

#### Hybrid fusion

The hybrid fusion method can leverage the strengths of both early and late fusion, potentially leading to improved performance. First, through Model1, the $n$ modal entity objects $(x_{1} ,x_{2} , \ldots ,x_{n}$) are taken as input to obtain the preliminary decision result, and then the decision result is integrated with the decision result of other unimodal models include $k - n$-th modal to obtain the final prediction output. This fusion method can be expressed as Eq. (3) and (4):

4 $${y = D\left( {d\left( {x_{1} ,x_{2} , \ldots ,x_{n} } \right), d\left( {x_{n + 1} } \right), \ldots , d\left( {x_{k} } \right)} \right)},$$

5 $${d\left( {x_{i} } \right) = M_{i} \left( {f\left( {x_{i} } \right)} \right)},$$

where $x_{i}$ denotes *i*-th data source, $f\left( {x_{i} } \right)$ represents the feature extracted by the model, $d(x_{i} )$ represents the decision judgment made by the model $M_{i}$, and $D$ represents the comprehensive decision method.

### The foreland of methodology and application for pathogenomics integration

Prognosis refers to the prospect of recovery as anticipated from the usual course of disease or peculiarities of the case, such as the probability of the patient's tumor recurrence, distant metastasis, or death. Risk stratification can be accomplished using in the tumor, nodes, and metastases (TNM) staging system, molecular features or clinical variables. However, ameliorating prognostic insight is an active area of research with frontiers in survival modeling, including multimodal and pan-cancer approaches generally. Table 2 gives an overview of research works that harness pathomics and genomics for multimodal fusion to apply in clinical prediction tasks.

**Table 2:** Summarizes the representative multimodal fusion works that combine pathomics and genomics for better clinical prediction tasks

Tumor type	Models	References	Data modality	Performance
Pan-cancer	Resnet-50; direct fusion on patch-level	Schmauch et al. [39]	RNA-seq and WSI	MSI status prediction, AUC: 0.81 (RNA + WSI)
PRAD	Resnet-50; autoencoder fusion in combined late space	Tan et al. [15]	RNA-seq and WSI	Malignant/benign tissue recognition, AUC: 0.74 (RNA + WSI)
BRCA	DenseNet-121; direct fusion on patch-level	He et al. [7]	RNA-seq and WSI	Subtype prediction, AUC: 0.83 ± 0.05 (RNA + WSI)
KIRC	Customized architecture; multivariate feature selection	Ning et al. [82]	RNA-seq and WSI	Survival prediction, C-index:0.79 (0.73–0.86) (RNA + WSI)
KIRC, GBM	VGG19; multimodal tensor fusion	Chen et al. [5]	RNA-seq, CNV, gene mutation and WSI	Survival prediction, KIRC C-index: 0.72 ± 0.031 (RNA + WSI); GBM C-index: 0.82 ± 0.010 (RNA + WSI)
Pan-cancer	SqueezeNet; unsupervised compression in a single feature vector	Cheerla et al. [63]	Clinical data, WSI, miRNA and mRNA	Survival prediction, C-index: 0.78 (miRNA + mRNA + WSI)
ER-BC	ResNet-101; canonical correction analysis	Xu et al. [83]	RNA-seq and WSI	Cancer-specific prediction, P-value 7.23e−06 (gene + WSI)
GBM	Customized architecture; two-stage feature aggregation	Hao et al. [84]	RNA-seq and WSI	Survival prediction, C-index: 0.70 ± 0.029 (RNA + WSI)
LIHC	Two-stage feature aggregation	Zhan et al. [85]	WSI and RNA-seq	Survival prediction, C-index: 0.75 (RNA + WSI)
KIRC, LIHC, LUAD	PCA; shared representation learning	Ning et al. [86]	WSI and RNA-seq	Survival prediction, C-index: 0.658–0.685 (WSI + RNA)

*DL* deep learning, *PRAD* prostate cancer, *BRCA* breast cancer, *GBM* glioblastoma, *ER-BC* estrogen receptor-positive breast cancer, *RNA-seq* RNA-sequencing, *CNVs* copy number variations, *WSI* whole slide image, *MSI* microsatellite, *AUC* the area under curves, *mRNA* messenger-RNA, *miRNA* micro-RNA, *ATAC-seq* assay for transposase-accessible chromatin using sequencing, *TCGA* The Cancer Genome Atlas, *TCIA* The Cancer Imaging Archive, *GDC* Genomic Data Commons portal, *C-index* concordance index, *PCA* principal components analysis, *KIRC* kidney renal clear cell carcinoma, *LIHC* liver hepatocellular carcinoma, *LUAD* lung adenocarcinoma

The main goal of multimodal fusion technology is to narrow the distribution gap in semantic subspace while maintaining the integrity of modal-specific semantics. Meanwhile, the key of multimodal fusion architecture is to implement feature concatenation. One of prevailing multimodal learning applications is based on Autoencoder (AE) models [60], providing DL models with capabilities to integrate different data modalities into a single end-to-end optimized model [61]. AE architecture usually starts with encoding each input modality into a representation vector of lower dimension, followed by a feature combination step to aggregate these vectors together, which comprises an encoder and a decoder working in tandem [62]. For instance, Tan et al. [15] developed SpaCell based on AE models to integrate millions of pixel intensity values with thousands of gene expression measurements from spatially barcoded spots in pathological tissue. This approach showed better performance than unimodal method alone.

Feature vector concatenation is also a common straightforward strategy for integrating pathomics and genomics [48, 63–65]. Shao [66] proposed an ordinal multi-modal feature selection (OMMFS) method that identified important features from each modality with the consideration of the intrinsic relationship between modalities. Chen [5] introduced a sophisticated end-to-end integrated late fusion framework for fusing the learned deep features from histology images, at patch-level and cell graph-level, and learned genomic features from genomic profiles. Pairwise feature interactions across modalities by taking the Kronecker product of unimodal feature representations and gating attention mechanism, were used to construct prognostic models for glioma and Clear Cell Renal Cell Carcinoma (CCRCC). Cheerla [63] constructed a deep learning-based pan-cancer model with auto encoder to exact four data modalities (gene expression, miRNA data, clinical data, and WSI) into a single feature vector for each patient, handing missing data through a resilient, multimodal dropout method, to predict survival of patients. These studies above showed combination of WSI and genomic data and found that the model performance outperformed than unimodal, while survival model is also suitable for computational modeling at the pan-cancer level. Wulczyn et al. [67] trained survival models for 10 cancer types and evaluated the predictive performance of their model in each cancer type. Vale-silva et al. [51] trained pan-cancer and multimodal models across 33 cancer types. The current consensus seems to be that histopathology-based feature can facilitate survival patterns by using genomic or clinical variables. However, the ultimate application in clinical setting may depend heavily on the selective image features, model type and well-curated datasets, among other factors.

Deep learning approaches could capture different perspectives of tumor morphology, but for a successful model to translate into new insights, it is critical to disambiguate tissue types to comprehend model predictions. Tissue type area ratio [30] and connectivity [68] will affect the final prediction results. The morphology of the intratumor stroma may be a stronger prognostic indicator than the tumor itself [69, 70]. Furthermore, the loss function adapted to the correct checking nature of the survival data outperforms a single binary classifier. However, multi-task approaches that combine multiple binary classifiers or survival loss with binary classifiers may yield better risk stratification.

In general, the studies discussed above demonstrate that multimodal fusion strategies with histopathological images and genomic profiles improve clinical prediction and patient stratification over digital pathology and molecular methods alone. However, the improvements observed in these early studies ought to be confirmed by adequate statistical analysis and external validation. Further experiments are indispensable to demonstrate the performance of generalizability and robustness to apply these approaches in real clinical settings. As for transcriptomic studies, much higher sample sizes are needed to make broader conclusions from the experimental reports. Besides, large language models (LLMs) [71, 72] have exhibited exceptional performance in the field of natural language processing, various general models similar to ChatGPT [71] have been developed in multi-omics tasks, such as scBERT [73], Geneformer [74], SIMBA [75] and scDesign3 [76], which can realize cell type annotation, network dynamics predictions, gene regulation, single cell simulation, etc. generic tasks in genomics. There is also work on segment anything model (SAM) [77]-based general tools for segmenting medical images [78] in clinical diagnosis. The latest research is that MI-zero [79] developed by Mahmood lab to realize the general classification of pathological images and ClinicalGPT [80] developed by BUPT and unified multimodal transformer-based models [81] related to general medical diagnosis. However, there is currently no overarching LLM for pathogenomics, indicating the great opportunities for future growth in this field.

## Challenges and opportunities in clinical adoption

In summary, the application of artificial intelligence in pathogenomics has demonstrated exceptional capabilities in the prediction of gene mutations, cancer subtypes, stratification, and prognosis. This has significantly contributed to the theoretical foundations for precise diagnosis, treatment, and prognosis of tumors. However, the integration of quantitative measurements from multi-modality data for clinical prognostication remains a formidable challenge. This is largely due to the high dimensionality and heterogeneity of the data.

### Multi-source, complex and unstandardized datasets in data availability

It is increasingly recognized that many cancer characteristics have an impact on the prognosis of cancer patients, including genomics, proteomics, clinical parameters, and invasive biomarkers of tumors. Perhaps the greatest challenge in multimodal machine learning is data scarcity in multi-source, complex and unstandardized datasets [12, 87].

The performance of any AI-based approach depends primarily on the quantity and quality of input data. The data used to train the AI algorithm needs to be clean, carefully collected and curated, have a maximum signal-to-noise ratio, and be as accurate and comprehensive as possible to achieve maximum predictive performance. When harnessing complex, unstandardized datasets from multicenter sources, the availability of datasets played a crucial role in the next process. Stained tissue specimens are often manually located and scanned, with limited clinical annotations and tremendous storage requirements. While AI algorithms [88–90] have been developed to standardize data, including staining and color normalization techniques. In recent years, studies have [91, 92] also been devoted to establishing comprehensive quality control and standardization tools, providing useful insights for preprocessing heterogeneous and multicenter datasets.

Developing and implementing the multi-modal fusion model requires access to matched pathology, genomic data, and clinical data. Such cross-institutional data sharing is essential to promote and test model generalizability. However, most medical datasets are still too sparse to be useful for the training of advanced machine learning techniques, and how to overcome these challenges is urgent to be solved. Leading platforms include the database of Genotypes and Phenotypes (dbGaP), the European Genome-phenome Archive (EGA), The Cancer Imaging Archive (TCIA), the Genomic Data Commons (GDC), and other resources in the National Cancer Institute (NCI) Cancer Research Data Commons. Beyond matched genomic data and H&E WSIs of TCGA and the Molecular Taxonomy of Breast Cancer International Consortium (METABRIC), public resources contain only small patient cohorts with multiple data modalities (IvyGAP [17]). To achieve this on a large scale, the exciting news is that Genomics England and the UK National Pathology Imaging Co-operative (NPIC [3]) announced a new initiative, the Genomics Pathology Imaging Collection (GPIC), combining digital pathology and genomic data to create a unique multi-omic resource for cancer research. This collaboration builds on the rich data of the 100,000 Genomes Project to add over 250,000 additional high-resolution WSI alongside matched structured pathology reports, somatic and germline sequence data, radiology data, and longitudinal clinical data in the Genomics England Trusted Research Environment. GPIC as a unique pathomic dataset of world-leading scale and quality, enables the next generation of AI for clinical cancer diagnostics, treatment and prognosis, greatly alleviating the challenge of public data scarcity.

On the other hand, federated learning [93] is a potential solution for the logistical challenges of anonymizing data and institutional privacy policies, especially via decentralized dataset distillation in resource-constrained edge environments [94]. Depending on the choice of model, federated learning may require novel training methods but enables training on multi-institutional cohorts without data leaving the local network [95, 96].

### Lack of repeatability and reproducibility in clinical settings

Repeatability and benchmarking are one of the major challenges of AI, and many published biomedical AI studies fail to provide source code, test data, or both. A key reason to try to validate independently with separate test sets is to ensure that these methods are resilient to pre-analysis sources of variation, including WSI preparation, scanner Models, and protocols.

To foster transparency, scientific repeatability, and measurable development, researchers should be encouraged to place new intermodal architectures and preprocessing solutions in standard repositories [97, 98] such as ModelHub.ai, github.com. and commercial vendors like Amazon S3, open-source products, Delta Lake for instance.

Meanwhile, due to the variability of the convolution kernel of the model, the overfitting or underfitting of the training data, etc., the identification of imaging biomarkers related to the prognosis from research will be irreproducible. Multimodal machine learning (ML) is more prone to overfitting [99, 100], because in most cases, the multimodal dataset is smaller and the multimodal model needs to fit more parameters. Traditional ML models enable investigators to calculate the required dataset size for a tolerable generalization error prior to analytical workflows. In addition to center-specific confounders, the actual clinical setting has unpredictable effects on model performance, often resulting in substantial performance degradations.

Multimodal ML models should be used judiciously for tasks with large statistical sample sizes and with strategies to combat overfittings, such as early stopping, data augmentation, gradient blending, weight decay, hard and soft sharing of hidden layer parameters. Investigators must be wary of spurious results due to institutional biases and small sizes, with cross-validation, retrospective external validation, prospective validation and clinical trials serving as key measures to assess algorithm effectiveness.

### Interpretability of models for trustworthy AI

Comprehension of how abstracted features from different modalities affect the model's inference remains another significant problem, while the exploration of interpretability of ML models, making the deep learning model, treated as a “black box”, in a more trustworthy way [101, 102].

Despite their high accuracy and ease of applicability, the lack of interpretability and contrasting domain-inspired intuitive criticism in handcrafted networks is a possible potential obstacle to the clinical application of deep neural networks. Hand-crafted feature-based AI approaches in histological images can provide better interpretability because they are often developed in conjunction with domain experts since the features were pre-fined, either in a domain-agnostic [101, 103, 104] or domain-inspired [105] manner. However, creating bespoke hand-crafted features is often a challenging and trivial task due to the considerable time and domain knowledge that pathologists or oncologists have invested in developing this method. This could critically impact the trustworthiness of model performance.

Interpretation of extracted features hinders the development of multimodal fusion studies to a certain degree. Computational fusion methods require not only the consideration of the discriminative power of the extracted features in the task but also the interpretability of these features. Focused efforts to clarify the concept within medicine have shown that clinicians generally view interpretability as transparency in model reasoning, adjustable features, and limitations [106].

**Fig. 3:**
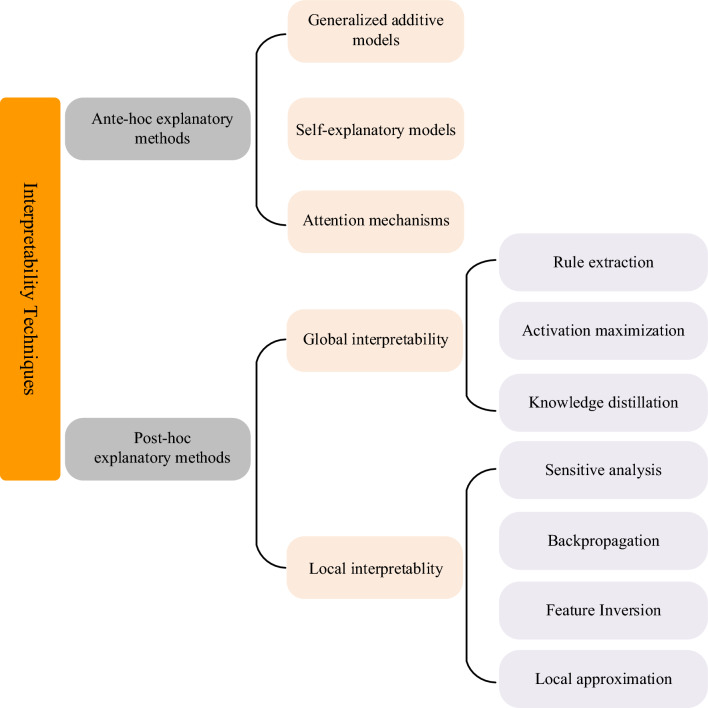
Classification of feature-based interpretability methods. Generally, it can be divided into ante-hoc explanatory methods and post-hoc explanatory methods

The field of multimodal in biomedicine particularly stands to benefit from interpretable AI, both in terms of imaging patterns of clinical features and molecular expression profiles of disease. Towards this end, we summarize various interpretability techniques for intuitive classification, organizing them according to two fundamental characteristics: ante-hoc explanatory methods, where the target models incorporate an explanation module into their architecture so that they are capable of explaining their predictions; post-hoc explanatory methods, where aim to explain already trained and fixed target models. In this pathogenomics review, we mainly focus on feature-based explanations. As for feature-based post-hoc explanatory methods, LRP [107, 108], LIME [109], DeepLIFT [110], SHAP [111], Integrated Gradients [109], L2X [112], Anchors [113], Grad-CAM [114] and LS-Tree [115] are currently the most widespread form of explanatory techniques; while self-explanatory methods with feature-based explanations include RCNN [116], CAR [117], INVASE [118], as well as the influential class of attention models [119] are commonly used for common features like super-pixels for images as well. Figure 3 shows the classification of interpretability techniques, and structured representation of the varied categories of interpretability methods shown in this review.

In the realm of ante-hoc interpretability, the primary advantages lie in intrinsic transparency and credibility. This approach integrates explicability into the design and training process of the model, enabling concurrent predictions and explanations. However, its limitations include potentially increased complexity in model design and the possibility that an excessive focus on interpretability might compromise performance. Balancing model accuracy with interpretability poses a significant challenge in ante-hoc interpretability research and represents a crucial direction for future advancements in explainable AI research [120, 121]. Regarding post-hoc interpretability, its strengths are flexibility and wide applicability. This approach is adaptable to various types and complexities of models without necessitating architectural modifications. However, a notable downside is the potential for misconceptions. The evaluative challenge lies in devising methods that faithfully represent the decision-making model, mitigating inconsistencies between the explanations and the model's actual behaviors. This ensures the reliability and safety of the interpretative outcomes [120, 121]. In summary, within pathogenomics research, ante-hoc explanations provide profound insights, especially beneficial for relatively straightforward models or those require high levels of explicability from its inception, particularly in multimodal fusion analyses. Conversely, post-hoc interpretations provide flexibility and practicality in pathogenomics correlation and fusion analysis models, especially for complex and already-developed models.

We believe that researchers should comprehend learning models from a biological and clinical perspectives to facilitate reasonable multimodal implementation. Understanding a model is as crucial as enhancing its predictive power and can lead to greater mechanistic insight and testable hypotheses.

## Conclusions

We summarize the current state of pathogenomics, combining synthesized complementary modalities of data with emerging multimodal AI-driven approaches for better comprehension of diagnostic, prognostic, and predictive decision-making for oncology. One future direction of pathogenomics is the exploration of more “omics” techniques (transcriptomics, proteomics, metabolomics, etc.) combined with functional imaging data (such as perfusion, diffusion imaging and spectroscopy, etc.) to open up more new avenues for multidimensional pathogenomics via LLMs techniques. In conclusion, with further in-depth research, pathogenomics will play a more active role in the medical field, especially in cancer research, and is likely to revolutionize the diagnosis, treatment and prognosis progress of cancer patients via taking advantage of complementary information in an intuitive manner, and ultimately open novel perspectives for precision oncology and empower a healthy and productive life in the coming decade.

## Data Availability

Not applicable.

## Abbreviations

AE: Autoencoder
AI: Artificial intelligence
ATAC-seq: Assay for transposase-accessible chromatin using sequencing
AUC: Area under curves
BRCA: Breast cancer
BUPT: Beijing University of Posts and Telecommunications
CAMS: The Chinese Academy of Medical Sciences, China
CCRCC: Clear Cell Renal Cell Carcinoma
CE-CT: Contrast-enhanced computed tomography
C-index: Concordance index
CNN: Convolutional neural network
CNV: Copy number variation
COAD: Colon adenocarcinoma
CT: Computed tomography
dbGaP: Database of Genotypes and Phenotypes
DL: Deep learning
ECM: Extracellular matrix
EGA: The European Genome-phenome Archive
ER-BC: Estrogen receptor-positive breast cancer
GCN: Graph convolutional network
GDC: The Genomic Data Commons
GO: Gene Ontology
GPIC: The Genomics Pathology Imaging Collection
H&E: Hematoxylin–Eosin staining
ICB: Immune checkpoint blockade
IHC: Immunohistochemistry
KEGG: Kyoto Encyclopedia of Genes and Genomes
KIRC: Kidney renal clear cell carcinoma
LIHC: Liver hepatocellular carcinoma
LLMs: Large language models
LR: Logistic regression
LUAD: Lung adenocarcinoma
METABRIC: Molecular Taxonomy of Breast Cancer International Consortium
ML: Machine learning
mRNA: Messenger-RNA
miRNA: Micro-RNA
MSI: Microsatellite instability
NCI: National Cancer Institute
NGS: Next-generation sequencing
NLST: The National Lung Screening Trial
NPIC: The UK National Pathology Imaging Co-operative
OMMFS: Ordinal multi-modal feature selection
PCA: Principal components analysis
READ: Rectum adenocarcinoma
RF: Random forest
PRAD: Prostate cancer
RNA-seq: RNA sequencing
SAM: Segment anything model
SPORE: The University of Texas Special Program of Research Excellence
STAD: Stomach cancer
SVM: Support vector machines
TCGA: The Cancer Genome Atlas
TCIA: The Cancer Imaging Archive
TIL: Tumor-infiltrating lymphocyte
TME: Tumor microenvironment
TNBC: Triple-negative breast cancer
TNM: Tumor, nodes, and metastases
WSI: Whole slide image

## References

[1] Sung H, Ferlay J, Siegel RL, Laversanne M, Soerjomataram I, Jemal A, Bray F (2021). Global cancer statistics 2020: GLOBOCAN estimates of incidence and mortality worldwide for 36 cancers in 185 countries. CA Cancer J Clin.

[2] Bera K, Schalper KA, Rimm DL, Velcheti V, Madabhushi A (2019). Artificial intelligence in digital pathology—new tools for diagnosis and precision oncology. Nat Rev Clin Oncol.

[3] Jennings CN, Humphries MP, Wood S, Jadhav M, Chabra R, Brown C, Chan G, Kaye D, Bansal D, Colquhoun C (2022). Bridging the gap with the UK genomics pathology imaging collection. Nat Med.

[4] Bi XA, Hu X, Xie Y, Wu H (2021). A novel CERNNE approach for predicting Parkinson’s disease-associated genes and brain regions based on multimodal imaging genetics data. Med Image Anal.

[5] Chen RJ, Lu MY, Wang J, Williamson DFK, Rodig SJ, Lindeman NI, Mahmood F (2022). Pathomic fusion: an integrated framework for fusing histopathology and genomic features for cancer diagnosis and prognosis. IEEE Trans Med Imaging.

[6] Chen RJ, Lu MY, Williamson DFK, Chen TY, Lipkova J, Noor Z, Shaban M, Shady M, Williams M, Joo B, Mahmood F (2022). Pan-cancer integrative histology-genomic analysis via multimodal deep learning. Cancer Cell.

[7] He B, Bergenstråhle L, Stenbeck L, Abid A, Andersson A, Borg Å, Maaskola J, Lundeberg J, Zou J (2020). Integrating spatial gene expression and breast tumour morphology via deep learning. Nat Biomed Eng.

[8] Elemento O, Leslie C, Lundin J, Tourassi G (2021). Artificial intelligence in cancer research, diagnosis and therapy. Nat Rev Cancer.

[9] Diao JA, Wang JK, Chui WF, Mountain V, Gullapally SC, Srinivasan R, Mitchell RN, Glass B, Hoffman S, Rao SK (2021). Human-interpretable image features derived from densely mapped cancer pathology slides predict diverse molecular phenotypes. Nat Commun.

[10] Cheng S, Liu S, Yu J, Rao G, Xiao Y, Han W, Zhu W, Lv X, Li N, Cai J (2021). Robust whole slide image analysis for cervical cancer screening using deep learning. Nat Commun.

[11] Niazi MKK, Parwani AV, Gurcan MN (2019). Digital pathology and artificial intelligence. Lancet Oncol.

[12] Boehm KM, Khosravi P, Vanguri R, Gao J, Shah SP (2022). Harnessing multimodal data integration to advance precision oncology. Nat Rev Cancer.

[13] Fu Y, Jung AW, Torne RV, Gonzalez S, Vöhringer H, Shmatko A, Yates LR, Jimenez-Linan M, Moore L, Gerstung M (2020). Pan-cancer computational histopathology reveals mutations, tumor composition and prognosis. Nat Cancer.

[14] Kather JN, Heij LR, Grabsch HI, Loeffler C, Echle A, Muti HS, Krause J, Niehues JM, Sommer KAJ, Bankhead P (2020). Pan-cancer image-based detection of clinically actionable genetic alterations. Nat Cancer.

[15] Tan X, Su A, Tran M, Nguyen Q (2020). SpaCell: integrating tissue morphology and spatial gene expression to predict disease cells. Bioinformatics.

[16] Hanahan D, Coussens LM (2012). Accessories to the crime: functions of cells recruited to the tumor microenvironment. Cancer Cell.

[17] Puchalski RB, Shah N, Miller J, Dalley R, Nomura SR, Yoon JG, Smith KA, Lankerovich M, Bertagnolli D, Bickley K (2018). An anatomic transcriptional atlas of human glioblastoma. Science.

[18] Rao A, Barkley D, França GS, Yanai I (2021). Exploring tissue architecture using spatial transcriptomics. Nature.

[19] Schapiro D, Jackson HW, Raghuraman S, Fischer JR, Zanotelli VRT, Schulz D, Giesen C, Catena R, Varga Z, Bodenmiller B (2017). histoCAT: analysis of cell phenotypes and interactions in multiplex image cytometry data. Nat Methods.

[20] Somarakis A, Van Unen V, Koning F, Lelieveldt B, Hollt T (2021). ImaCytE: visual exploration of cellular micro-environments for imaging mass cytometry data. IEEE Trans Vis Comput Graph.

[21] Wu F, Fan J, He Y, Xiong A, Yu J, Li Y, Zhang Y, Zhao W, Zhou F, Li W (2021). Single-cell profiling of tumor heterogeneity and the microenvironment in advanced non-small cell lung cancer. Nat Commun.

[22] Lafarge MW, Koelzer VH (2021). Towards computationally efficient prediction of molecular signatures from routine histology images. Lancet Digit Health.

[23] Liang J, Zhang W, Yang J, Wu M, Dai Q, Yin H, Xiao Y, Kong L (2023). Deep learning supported discovery of biomarkers for clinical prognosis of liver cancer. Nat Mach Intell.

[24] Sirinukunwattana K, Domingo E, Richman SD, Redmond KL, Blake A, Verrill C, Leedham SJ, Chatzipli A, Hardy C, Whalley CM (2021). Image-based consensus molecular subtype (imCMS) classification of colorectal cancer using deep learning. Gut.

[25] Skrede OJ, De Raedt S, Kleppe A, Hveem TS, Liestøl K, Maddison J, Askautrud HA, Pradhan M, Nesheim JA, Albregtsen F (2020). Deep learning for prediction of colorectal cancer outcome: a discovery and validation study. Lancet.

[26] Yu G, Sun K, Xu C, Shi XH, Wu C, Xie T, Meng RQ, Meng XH, Wang KS, Xiao HM, Deng HW (2021). Accurate recognition of colorectal cancer with semi-supervised deep learning on pathological images. Nat Commun.

[27] Ehteshami Bejnordi B, Veta M, Johannes van Diest P, van Ginneken B, Karssemeijer N, Litjens G, van der Laak J, Hermsen M, Manson QF, Balkenhol M (2017). Diagnostic assessment of deep learning algorithms for detection of lymph node metastases in women with breast cancer. JAMA.

[28] Lotter W, Diab AR, Haslam B, Kim JG, Grisot G, Wu E, Wu K, Onieva JO, Boyer Y, Boxerman JL (2021). Robust breast cancer detection in mammography and digital breast tomosynthesis using an annotation-efficient deep learning approach. Nat Med.

[29] Kuntz S, Krieghoff-Henning E, Kather JN, Jutzi T, Höhn J, Kiehl L, Hekler A, Alwers E, von Kalle C, Fröhling S (2021). Gastrointestinal cancer classification and prognostication from histology using deep learning: systematic review. Eur J Cancer.

[30] Wang X, Chen Y, Gao Y, Zhang H, Guan Z, Dong Z, Zheng Y, Jiang J, Yang H, Wang L (2021). Predicting gastric cancer outcome from resected lymph node histopathology images using deep learning. Nat Commun.

[31] Campanella G, Hanna MG, Geneslaw L, Miraflor A, Werneck Krauss Silva V, Busam KJ, Brogi E, Reuter VE, Klimstra DS, Fuchs TJ (2019). Clinical-grade computational pathology using weakly supervised deep learning on whole slide images. Nat Med.

[32] Pinckaers H, Bulten W, van der Laak J, Litjens G (2021). Detection of prostate cancer in whole-slide images through end-to-end training with image-level labels. IEEE Trans Med Imaging.

[33] Coudray N, Ocampo PS, Sakellaropoulos T, Narula N, Snuderl M, Fenyö D, Moreira AL, Razavian N, Tsirigos A (2018). Classification and mutation prediction from non-small cell lung cancer histopathology images using deep learning. Nat Med.

[34] Lu MY, Williamson DFK, Chen TY, Chen RJ, Barbieri M, Mahmood F (2021). Data-efficient and weakly supervised computational pathology on whole-slide images. Nat Biomed Eng.

[35] Wang X, Chen H, Gan C, Lin H, Dou Q, Tsougenis E, Huang Q, Cai M, Heng PA (2020). Weakly supervised deep learning for whole slide lung cancer image analysis. IEEE Trans Cybern.

[36] Bhinder B, Gilvary C, Madhukar NS, Elemento O (2021). Artificial intelligence in cancer research and precision medicine. Cancer Discov.

[37] Echle A, Grabsch HI, Quirke P, van den Brandt PA, West NP, Hutchins GGA, Heij LR, Tan X, Richman SD, Krause J (2020). Clinical-grade detection of microsatellite instability in colorectal tumors by deep learning. Gastroenterology.

[38] Kather JN, Pearson AT, Halama N, Jäger D, Krause J, Loosen SH, Marx A, Boor P, Tacke F, Neumann UP (2019). Deep learning can predict microsatellite instability directly from histology in gastrointestinal cancer. Nat Med.

[39] Schmauch B, Romagnoni A, Pronier E, Saillard C, Maillé P, Calderaro J, Kamoun A, Sefta M, Toldo S, Zaslavskiy M (2020). A deep learning model to predict RNA-Seq expression of tumours from whole slide images. Nat Commun.

[40] Luo X, Yin S, Yang L, Fujimoto J, Yang Y, Moran C, Kalhor N, Weissferdt A, Xie Y, Gazdar A (2019). Development and validation of a pathology image analysis-based predictive model for lung adenocarcinoma prognosis—a multi-cohort study. Sci Rep.

[41] Boehm KM, Aherne EA, Ellenson L, Nikolovski I, Alghamdi M, Vázquez-García I, Zamarin D, Roche KL, Liu Y, Patel D (2022). Multimodal data integration using machine learning improves risk stratification of high-grade serous ovarian cancer. Nat Cancer.

[42] Levy-Jurgenson A, Tekpli X, Kristensen VN, Yakhini Z (2020). Spatial transcriptomics inferred from pathology whole-slide images links tumor heterogeneity to survival in breast and lung cancer. Sci Rep.

[43] Poirion OB, Jing Z, Chaudhary K, Huang S, Garmire LX (2021). DeepProg: an ensemble of deep-learning and machine-learning models for prognosis prediction using multi-omics data. Genome Med.

[44] Cancer Genome Atlas Research Network (2011). Integrated genomic analyses of ovarian carcinoma. Nature.

[45] Kim S, Kim K, Choe J, Lee I, Kang J (2020). Improved survival analysis by learning shared genomic information from pan-cancer data. Bioinformatics.

[46] Savage RS, Yuan Y (2016). Predicting chemoinsensitivity in breast cancer with 'omics/digital pathology data fusion. R Soc Open Sci.

[47] Saltz J, Gupta R, Hou L, Kurc T, Singh P, Nguyen V, Samaras D, Shroyer KR, Zhao T, Batiste R (2018). Spatial organization and molecular correlation of tumor-infiltrating lymphocytes using deep learning on pathology images. Cell Rep.

[48] Mobadersany P, Yousefi S, Amgad M, Gutman DA, Barnholtz-Sloan JS, Velázquez Vega JE, Brat DJ, Cooper LAD (2018). Predicting cancer outcomes from histology and genomics using convolutional networks. Proc Natl Acad Sci USA.

[49] Courtiol P, Maussion C, Moarii M, Pronier E, Pilcer S, Sefta M, Manceron P, Toldo S, Zaslavskiy M, Le Stang N (2019). Deep learning-based classification of mesothelioma improves prediction of patient outcome. Nat Med.

[50] Zhong T, Wu M, Ma S (2019). Examination of independent prognostic power of gene expressions and histopathological imaging features in cancer. Cancers.

[51] Vale-Silva LA, Rohr K (2021). Long-term cancer survival prediction using multimodal deep learning. Sci Rep.

[52] Liu Y, Yang M, Deng Y, Su G, Enninful A, Guo CC, Tebaldi T, Zhang D, Kim D, Bai Z (2020). High-spatial-resolution multi-omics sequencing via deterministic barcoding in tissue. Cell.

[53] Niu Y, Wang L, Zhang X, Han Y, Yang C, Bai H, Huang K, Ren C, Tian G, Yin S (2022). Predicting tumor mutational burden from lung adenocarcinoma histopathological images using deep learning. Front Oncol.

[54] Ding K, Zhou M, Wang H, Zhang S, Metaxas DN (2022). Spatially aware graph neural networks and cross-level molecular profile prediction in colon cancer histopathology: a retrospective multi-cohort study. Lancet Digit Health.

[55] Zheng H, Momeni A, Cedoz PL, Vogel H, Gevaert O (2020). Whole slide images reflect DNA methylation patterns of human tumors. NPJ Genom Med.

[56] Acosta JN, Falcone GJ, Rajpurkar P, Topol EJ (2022). Multimodal biomedical AI. Nat Med.

[57] Ngiam J, Khosla A, Kim M, Nam J, Lee H, Ng AY. Multimodal deep learning. In: Proceedings of the 28th international conference on international conference on machine learning. Bellevue: Omnipress; 2011. p. 689–96.

[58] Atrey PK, Hossain MA, El Saddik A, Kankanhalli MS (2010). Multimodal fusion for multimedia analysis: a survey. Multimed Syst.

[59] Nojavanasghari B, Gopinath D, Koushik J, Baltrušaitis T, Morency L-P. Deep multimodal fusion for persuasiveness prediction. In: Proceedings of the 18th ACM international conference on multimodal interaction. Tokyo: Association for Computing Machinery; 2016. p. 284–8.

[60] Gao J, Li P, Chen Z, Zhang J (2020). A survey on deep learning for multimodal data fusion. Neural Comput.

[61] Eraslan G, Avsec Ž, Gagneur J, Theis FJ (2019). Deep learning: new computational modelling techniques for genomics. Nat Rev Genet.

[62] Tschannen M, Bachem O, Lucic M. Recent advances in autoencoder-based representation learning. arXiv preprint. 2018. arXiv:1812.05069.

[63] Cheerla A, Gevaert O (2019). Deep learning with multimodal representation for pancancer prognosis prediction. Bioinformatics.

[64] Cheng J, Zhang J, Han Y, Wang X, Ye X, Meng Y, Parwani A, Han Z, Feng Q, Huang K (2017). Integrative analysis of histopathological images and genomic data predicts clear cell renal cell carcinoma prognosis. Cancer Res.

[65] Ren J, Karagoz K, Gatza ML, Singer EA, Sadimin E, Foran DJ, Qi X (2018). Recurrence analysis on prostate cancer patients with Gleason score 7 using integrated histopathology whole-slide images and genomic data through deep neural networks. J Med Imaging.

[66] Shao W, Han Z, Cheng J, Cheng L, Wang T, Sun L, Lu Z, Zhang J, Zhang D, Huang K (2020). Integrative analysis of pathological images and multi-dimensional genomic data for early-stage cancer prognosis. IEEE Trans Med Imaging.

[67] Wulczyn E, Steiner DF, Xu Z, Sadhwani A, Wang H, Flament-Auvigne I, Mermel CH, Chen PC, Liu Y, Stumpe MC (2020). Deep learning-based survival prediction for multiple cancer types using histopathology images. PLoS ONE.

[68] Abbet C, Zlobec I, Bozorgtabar B, Thiran J-P. Divide-and-rule: self-supervised learning for survival analysis in colorectal cancer. In: International conference on medical image computing and computer-assisted intervention. Springer; 2020. p. 480–9.

[69] Bhargava HK, Leo P, Elliott R, Janowczyk A, Whitney J, Gupta S, Fu P, Yamoah K, Khani F, Robinson BD (2020). Computationally derived image signature of stromal morphology is prognostic of prostate cancer recurrence following prostatectomy in African American patients. Clin Cancer Res.

[70] Beck AH, Sangoi AR, Leung S, Marinelli RJ, Nielsen TO, van de Vijver MJ, West RB, van de Rijn M, Koller D (2011). Systematic analysis of breast cancer morphology uncovers stromal features associated with survival. Sci Transl Med.

[71] Brown T, Mann B, Ryder N, Subbiah M, Kaplan JD, Dhariwal P, Neelakantan A, Shyam P, Sastry G, Askell A (2020). Language models are few-shot learners. Adv Neural Inf Process Syst.

[72] Radford A, Wu J, Child R, Luan D, Amodei D, Sutskever I. Language models are unsupervised multitask learners. OpenAI blog. 2019.

[73] Yang F, Wang W, Wang F, Fang Y, Tang D, Huang J, Lu H, Yao J (2022). scBERT as a large-scale pretrained deep language model for cell type annotation of single-cell RNA-seq data. Nat Mach Intell.

[74] Theodoris CV, Xiao L, Chopra A, Chaffin MD, Al Sayed ZR, Hill MC, Mantineo H, Brydon EM, Zeng Z, Liu XS, Ellinor PT (2023). Transfer learning enables predictions in network biology. Nature.

[75] Chen H, Ryu J, Vinyard ME, Lerer A, Pinello L (2023). SIMBA: single-cell embedding along with features. Nat Methods.

[76] Song D, Wang Q, Yan G, Liu T, Sun T, Li JJ (2023). scDesign3 generates realistic in silico data for multimodal single-cell and spatial omics. Nat Biotechnol.

[77] Kirillov A, Mintun E, Ravi N, Mao H, Rolland C, Gustafson L, Xiao T, Whitehead S, Berg AC, Lo W-Y. Segment anything. arXiv preprint. 2023. arXiv:2304.02643.

[78] Deng R, Cui C, Liu Q, Yao T, Remedios LW, Bao S, Landman BA, Wheless LE, Coburn LA, Wilson KT. Segment anything model (SAM) for digital pathology: assess zero-shot segmentation on whole slide imaging. arXiv preprint. 2023. arXiv:2304.04155. 10.2352/EI.2025.37.14.COIMG-132 PMC11971729 40190816

[79] Lu MY, Chen B, Zhang A, Williamson DF, Chen RJ, Ding T, Le LP, Chuang Y-S, Mahmood F. Visual language pretrained multiple instance zero-shot transfer for histopathology images. In: Proceedings of the IEEE/CVF conference on computer vision and pattern recognition. 2023. p. 19764–75.

[80] Wang G, Yang G, Du Z, Fan L, Li X. ClinicalGPT: large language models finetuned with diverse medical data and comprehensive evaluation. arXiv preprint. 2023. arXiv:2306.09968.

[81] Zhou H-Y, Yu Y, Wang C, Zhang S, Gao Y, Pan J, Shao J, Lu G, Zhang K, Li W (2023). A transformer-based representation-learning model with unified processing of multimodal input for clinical diagnostics. Nat Biomed Eng.

[82] Ning Z, Pan W, Chen Y, Xiao Q, Zhang X, Luo J, Wang J, Zhang Y (2020). Integrative analysis of cross-modal features for the prognosis prediction of clear cell renal cell carcinoma. Bioinformatics.

[83] Xu S, Lu Z, Shao W, Yu CY, Reiter JL, Feng Q, Feng W, Huang K, Liu Y (2020). Integrative analysis of histopathological images and chromatin accessibility data for estrogen receptor-positive breast cancer. BMC Med Genom.

[84] Hao J, Kosaraju SC, Tsaku NZ, Song DH, Kang M (2020). PAGE-Net: interpretable and integrative deep learning for survival analysis using histopathological images and genomic data. Pac Symp Biocomput.

[85] Zhan Z, Jing Z, He B, Hosseini N, Westerhoff M, Choi EY, Garmire LX (2021). Two-stage Cox-nnet: biologically interpretable neural-network model for prognosis prediction and its application in liver cancer survival using histopathology and transcriptomic data. NAR Genom Bioinform.

[86] Ning Z, Du D, Tu C, Feng Q, Zhang Y (2022). Relation-aware shared representation learning for cancer prognosis analysis with auxiliary clinical variables and incomplete multi-modality data. IEEE Trans Med Imaging.

[87] Liang W, Tadesse GA, Ho D, Li F-F, Zaharia M, Zhang C, Zou J (2022). Advances, challenges and opportunities in creating data for trustworthy AI. Nat Mach Intell.

[88] Anand D, Ramakrishnan G, Sethi A. Fast GPU-enabled color normalization for digital pathology. In: 2019 international conference on systems, signals and image processing (IWSSIP); 5–7 June 2019. 2019. p. 219–24.

[89] Boschman J, Farahani H, Darbandsari A, Ahmadvand P, Van Spankeren A, Farnell D, Levine AB, Naso JR, Churg A, Jones SJ (2022). The utility of color normalization for AI-based diagnosis of hematoxylin and eosin-stained pathology images. J Pathol.

[90] Tellez D, Litjens G, Bándi P, Bulten W, Bokhorst JM, Ciompi F, van der Laak J (2019). Quantifying the effects of data augmentation and stain color normalization in convolutional neural networks for computational pathology. Med Image Anal.

[91] Chen Y, Zee J, Smith A, Jayapandian C, Hodgin J, Howell D, Palmer M, Thomas D, Cassol C, Farris AB (2021). Assessment of a computerized quantitative quality control tool for whole slide images of kidney biopsies. J Pathol.

[92] Hosseini MS, Brawley-Hayes JAZ, Zhang Y, Chan L, Plataniotis K, Damaskinos S (2020). Focus quality assessment of high-throughput whole slide imaging in digital pathology. IEEE Trans Med Imaging.

[93] Rieke N, Hancox J, Li W, Milletarì F, Roth HR, Albarqouni S, Bakas S, Galtier MN, Landman BA, Maier-Hein K (2020). The future of digital health with federated learning. NPJ Digit Med.

[94] Song R, Liu D, Chen DZ, Festag A, Trinitis C, Schulz M, Knoll A. Federated learning via decentralized dataset distillation in resource-constrained edge environments. arXiv preprint. 2022. arXiv:2208.11311.

[95] Dayan I, Roth HR, Zhong A, Harouni A, Gentili A, Abidin AZ, Liu A, Costa AB, Wood BJ, Tsai CS (2021). Federated learning for predicting clinical outcomes in patients with COVID-19. Nat Med.

[96] Zhao Y, Yu G, Wang J, Domeniconi C, Guo M, Zhang X, Cui L (2022). Personalized federated few-shot learning. IEEE Trans Neural Netw Learn Syst.

[97] Armbrust M, Das T, Sun L, Yavuz B, Zhu S, Murthy M, Torres J, Hovell HV, Ionescu A, Łuszczak A (2020). Delta lake: high-performance ACID table storage over cloud object stores. Proc VLDB Endow.

[98] Zagan E, Danubianu M. Cloud DATA LAKE: the new trend of data storage. In: 2021 3rd international congress on human–computer interaction, optimization and robotic applications (HORA); 11–13 June 2021. 2021. p. 1–4.

[99] Li Z, Kamnitsas K, Glocker B (2021). Analyzing overfitting under class imbalance in neural networks for image segmentation. IEEE Trans Med Imaging.

[100] Mummadi SR, Al-Zubaidi A, Hahn PY (2018). Overfitting and use of mismatched cohorts in deep learning models: preventable design limitations. Am J Respir Crit Care Med.

[101] Rudin C (2019). Stop explaining black box machine learning models for high stakes decisions and use interpretable models instead. Nat Mach Intell.

[102] Thorsen-Meyer HC, Nielsen AB, Nielsen AP, Kaas-Hansen BS, Toft P, Schierbeck J, Strøm T, Chmura PJ, Heimann M, Dybdahl L (2020). Dynamic and explainable machine learning prediction of mortality in patients in the intensive care unit: a retrospective study of high-frequency data in electronic patient records. Lancet Digit Health.

[103] Chen X, Kuang T, Deng H, Fung SH, Gateno J, Xia JJ, Yap PT (2022). Dual adversarial attention mechanism for unsupervised domain adaptive medical image segmentation. IEEE Trans Med Imaging.

[104] Kather JN, Ghaffari Laleh N, Foersch S, Truhn D (2022). Medical domain knowledge in domain-agnostic generative AI. NPJ Digit Med.

[105] Corredor G, Wang X, Zhou Y, Lu C, Fu P, Syrigos K, Rimm DL, Yang M, Romero E, Schalper KA (2019). Spatial architecture and arrangement of tumor-infiltrating lymphocytes for predicting likelihood of recurrence in early-stage non-small cell lung cancer. Clin Cancer Res.

[106] Holzinger A, Biemann C, Pattichis CS, Kell DB. What do we need to build explainable AI systems for the medical domain? arXiv preprint. 2017. arXiv:1712.09923.

[107] Arras L, Montavon G, Müller K-R, Samek W. Explaining recurrent neural network predictions in sentiment analysis. ArXiv. 2017. arXiv:1706.07206.

[108] Bach S, Binder A, Montavon G, Klauschen F, Müller KR, Samek W (2015). On pixel-wise explanations for non-linear classifier decisions by layer-wise relevance propagation. PLoS ONE.

[109] Ribeiro MT, Singh S, Guestrin C. “Why should I trust you?”: explaining the predictions of any classifier. In: Proceedings of the 22nd ACM SIGKDD international conference on knowledge discovery and data mining. San Francisco: Association for Computing Machinery; 2016. p. 1135–44.

[110] Shrikumar A, Greenside P, Kundaje A. Learning important features through propagating activation differences. In: Doina P, Yee Whye T, editors. Proceedings of the 34th international conference on machine learning, vol. 70. Proceedings of Machine Learning Research: PMLR; 2017. p. 3145–53.

[111] Lundberg SM, Lee S-I. A unified approach to interpreting model predictions. ArXiv. 2017. arXiv:1705.07874.

[112] Chen J, Song L, Wainwright M, Jordan M. Learning to explain: an information-theoretic perspective on model interpretation. In: Jennifer D, Andreas K, editors. Proceedings of the 35th international conference on machine learning, vol. 80. Proceedings of Machine Learning Research: PMLR; 2018. p. 883–92.

[113] Ribeiro MT, Singh S, Guestrin C. Anchors: high-precision model-agnostic explanations. In: Proceedings of the AAAI conference on artificial intelligence. 2018.

[114] Selvaraju RR, Cogswell M, Das A, Vedantam R, Parikh D, Batra D. Grad-CAM: visual explanations from deep networks via gradient-based localization. In: 2017 IEEE international conference on computer vision (ICCV); 22–29 Oct. 2017. 2017. p. 618–26.

[115] Chen J, Jordan M. Ls-tree: model interpretation when the data are linguistic. In: Proceedings of the AAAI conference on artificial intelligence. 2020. p. 3454–61.

[116] Lei T, Barzilay R, Jaakkola T. Rationalizing neural predictions. arXiv preprint. 2016. arXiv:1606.04155.

[117] Chang S, Zhang Y, Yu M, Jaakkola T. A game theoretic approach to class-wise selective rationalization. In: Advances in neural information processing systems, vol. 32. 2019.

[118] Yoon J, Jordon J, van der Schaar M. INVASE: instance-wise variable selection using neural networks. In: International conference on learning representations. 2018.

[119] Bahdanau D, Cho K, Bengio Y. Neural machine translation by jointly learning to align and translate. arXiv preprint. 2014. arXiv:1409.0473.

[120] Murdoch WJ, Singh C, Kumbier K, Abbasi-Asl R, Yu B (2019). Definitions, methods, and applications in interpretable machine learning. Proc Natl Acad Sci USA.

[121] Molnar C. Interpretable machine learning. Lulu.com; 2020.

